# Circadian Rhythms and Redox State in Plants: Till Stress Do Us Part

**DOI:** 10.3389/fpls.2018.00247

**Published:** 2018-03-05

**Authors:** Carmela R. Guadagno, Brent E. Ewers, Cynthia Weinig

**Affiliations:** ^1^Department of Botany, University of Wyoming, Laramie, WY, United States; ^2^Program in Ecology, University of Wyoming, Laramie, WY, United States; ^3^Department of Molecular and Cellular Life Sciences, University of Wyoming, Laramie, WY, United States

**Keywords:** circadian rhythms, ROS, redox state, plant stress response, chlorophyll *a* fluorescence

## Abstract

A growing body of evidence demonstrates a significant relationship between cellular redox state and circadian rhythms. Each day these two vital components of plant biology influence one another, dictating the pace for metabolism and physiology. Diverse environmental stressors can disrupt this condition and, although plant scientists have made significant progress in re-constructing functional networks of plant stress responses, stress impacts on the clock-redox crosstalk is poorly understood. Inter-connected phenomena such as redox state and metabolism, internal and external environments, cellular homeostasis and rhythms can impede predictive understanding of coordinated regulation of plant stress response. The integration of circadian clock effects into predictive network models is likely to increase final yield and better predict plant responses to stress. To achieve such integrated understanding, it is necessary to consider the internal clock not only as a gatekeeper of environmental responses but also as a target of stress syndromes. Using chlorophyll fluorescence as a reliable and high-throughput probe of stress coupled to functional genomics and metabolomics will provide insights on the crosstalk across a wide range of stress severity and duration, including potential insights into oxidative stress response and signaling. We suggest the efficiency of photosystem II in light conditions (*F*_v_′*/F*_m_′) to be the most dynamic of the fluorescence variables and therefore the most reliable parameter to follow the stress response from early sensing to mortality.

## Introduction

The link between the circadian clock and oxygenic metabolism is likely to have originated with the rise in oxygen concentration 3 billion years ago, when early photosynthetic bacteria started to use water as an electron donor. Given the lack of redox systems and the loss of an endogenous clock in archaeal taxa living in the absence of oxygen, the co-evolution of cellular clockwork and aerobic metabolism seems extremely probable (Schippers et al., 2013). It is thus reasonable to expect the Great Oxidation Event would cause selection for organisms capable of respiring and/or evolving molecular oxygen, with the most successful organisms acquiring Reactive Oxygen Species (ROS) removal systems to avoid relegation to anaerobic niches (Edgar et al., 2012).

Although the clock/redox state relationship lasts for the entire life of a plant, our current understanding of it is very limited. We present current advances in the study of the clock/redox state association in plants with particular attention to the influence of environmental stressors on this dynamic duo. We advocate for the use of chlorophyll *a* fluorescence, not only to monitor the plant physiological status during and after stress, but also to gain relevant information on possible clock alterations caused by this disturbance.

## Plant Circadian Rhythms

All living things on Earth encounter daily oscillations in environmental factors. A diverse range of organisms has evolved an endogenous clock, which permits anticipation of predictable fluctuations in environmental conditions arising from the daily rotation of our planet (McClung, 2006), enabling organisms to coordinate the timing of biological processes with the environmental conditions (Pittendrigh, 1960). The clock may be entrained by diverse external inputs, providing circadian (*circa*, about, and *dian*, a day) rhythms with a periodicity of approximately 24-h (DeCoursey et al., 2000; Green et al., 2002), which persist under constant conditions (de Mairan, 1729; Salome and McClung, 2004; Harmer, 2009; McClung, 2011; Guerriero et al., 2012). The resonance between endogenous clock and exogenous cycles affects performance (Todd et al., 2003; Dodd et al., 2005; de Montaigu et al., 2015; de Montaigu and Coupland, 2017), as does quantitative (naturally occurring) clock variation (Salmela et al., 2016; Rubin et al., 2017). The clock can be differentially affected by external cues over 24-h. Based on the time of day, the *gating* property of the clock causes different amplitude in the transcriptional responses of clock-regulated genes to the same environmental stimulus (Wilkins et al., 2009, 2010; Grundy et al., 2015).

The clock drives temporal gene expression with physiological consequences, such as gas exchange, from individual to ecosystem scales (Resco de Dios and Gessler, 2017) and many aspects of plant development and its interactions with the environment (Dodd et al., 2005; Gibon et al., 2006; Khan et al., 2010; Edwards et al., 2011, 2012; Kerwin et al., 2011; Wulund and Reddy, 2015; Resco de Dios, 2017; Hubbard et al., 2018). At dawn, the clock enhances the resistance to oxidative species produced during the light-harvesting processes (Doherty and Kay, 2010), it is responsible for part of the signaling that governs stomata opening (Hotta et al., 2007), and it controls the mobilization of carbohydrates at dusk (Graf and Smith, 2011). The clock also causes hormonal waves influencing life-history traits, such as plant size at reproduction (Hanano et al., 2006) and floral development (Somers et al., 1998; Doyle et al., 2002; Song et al., 2012). Clock modulation for several hormones is characterized in the model organism *Arabidopsis thaliana*, and some of these hormones have been recently shown to mediate a response to changes in light/dark cycles for controlled environments (Nitschke et al., 2016). In *Arabidopsis*, about 30% of the transcriptome is clock-regulated in day/night growing conditions (Covington et al., 2008). This percentage rises to 80% in free running settings (Barak et al., 2000; Haydon et al., 2013; Nagel et al., 2015). This crucial role for the clock has been confirmed for other dicots (Ramos et al., 2005; Wilkins et al., 2009; Yon et al., 2012; Marcolino-Gomes et al., 2014); while metabolic rhythmicity has been shown in monocot crops (Filichkin et al., 2011; Calixto et al., 2015).

While they were originally viewed as mere clock outputs, metabolic oscillations have now been shown to feed back to the clock (Harmer, 2009; Schippers et al., 2013), resulting in a complex and fine-tuned cellular network (Gallego and Virshup, 2007; Pruneda-Paz and Kay, 2010; Sanchez et al., 2011). For instance, the circadian clock tunes both timing and capacity of sugar production *via* photosynthesis but, at the same time, sugar signals entrain the clock (Haydon et al., 2013). Importantly, plants’ nutritional status seems to feed back to the circadian clock, with the most relevant impacts for nitrogen and iron assimilation (Gutiérrez et al., 2008; Chen et al., 2013). Indeed nitrogen transporters are up regulated at night in drought stressed plants to counter-act lower water uptake rates (Greenham et al., 2017). Currently, redox mechanisms seem to be the best candidate to set the rhythms for this biochemical oscillator.

## Ros Homeostasis

Redox state indicates the balance of oxidized versus reduced forms of electron donors and acceptors in a cell. When plants interact with the surrounding environment, the redox state is determined mainly by sudden production of highly reactive molecules, trigging signaling at a systemic level (Woodson, 2016). Reactive Oxygen Species (ROS) are inevitable by-products of aerobic metabolism and electron transport processes. Plants produce ROS because of the electron-transfer to and from molecular oxygen taking place in chloroplasts, mitochondria, and plasma membranes (Baxter et al., 2014). Peroxisomes are also involved in oxygen metabolism as signal mediators, regulating an array of oxidases-catalases to maintain H_2_O_2_ balance (Bonekamp et al., 2009). These reactive molecules are harmful when their generation exceeds their elimination. ROS can extensively damage all biomolecules – from lipids to proteins to nucleic acids – possibly leading to cell death (Ahmad et al., 2008). However, the perpetuation of a ROS signal, from the site of stress origin to the target of response, is crucial for coping with environmental stimuli (Bailey-Serres and Mittler, 2006). Therefore, a complex network of enzymatic and non-enzymatic antioxidants continuously scavenges excess ROS: the activity of these scavengers *de facto* translates into ROS homeostasis at the cellular level under favorable conditions (Doherty and Kay, 2010; Gilroy et al., 2014; Kangasjärvi and Kangasjärvi, 2014).

A number of studies have shown that the redox state is both regulated by and acts as a feedback on the endogenous clock under several environmental conditions (Krishnan and Pereira, 2008; Stangherlin and Reddy, 2013; Milev et al., 2015). The presence of ubiquitous sinks for H_2_O_2_ (peroxiredoxin protein family) supports the hypothesis of prevailing interlinks among ROS, metabolic pathways and the clock (Edgar et al., 2012; Yon et al., 2012; Hoyle and O’Neill, 2015). Rhythmic oscillations in ROS production seem to be altered under stress, as a consequence of redox feedbacks; yet, several components of redox-signaling pathways in plant cells remain undescribed (Suzuki et al., 2012; Kangasjärvi and Kangasjärvi, 2014).

## Clock and Redox State: in Good Times and in Bad Times

The use of clock mutants has been critical in identifying circadian regulation of ROS homeostasis under several environmental conditions (Baxter et al., 2014; Greenham and McClung, 2015). ROS production and the activity of enzymatic scavengers have been shown to synchronously peak at specific times of the day (Lai et al., 2012). At the cellular level, a functioning clock directly regulates the redox state, coordinating the temporal activity of several scavengers. Fluctuations in one or multiple environmental factors affect the link between the clock and plant redox state eventually influencing growth, development and metabolism at the whole-plant level (Ahmad et al., 2008; Das and Roychoudhury, 2014; Gyöngyösi and Káldi, 2014).

**FIGURE 1:**
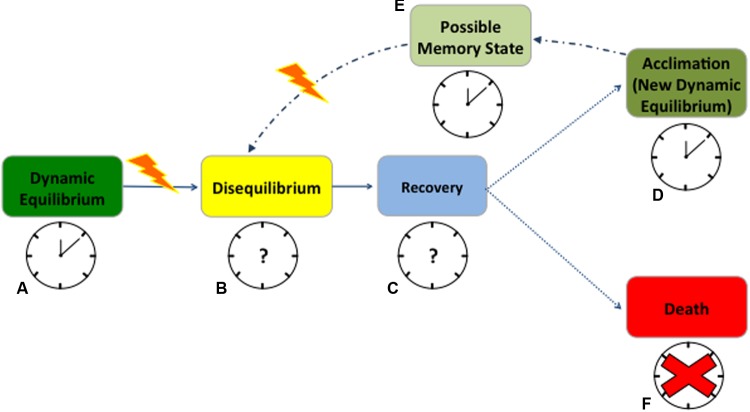
Anticipated redox state and circadian rhythms response to abiotic stress in plants. When cells are healthy, plants are in a dynamic equilibrium with the environment and the clock promptly resonates with environmental cycles **(A)**. At stress occurrence, changes in the redox state compromise cellular homeostasis, leading to a state of disequilibrium, with almost unknown consequences on the clock **(B)**. During a phase of recovery or strain, plants try to cope with the stress but how the clock behaves during this time is still uncertain **(C)**. A successful recovery will lead to a new dynamic equilibrium (acclimation) after stress exposure **(D)**. When acclimated, plants maintain a state of memory for which they will possibly experience fewer disturbances from the next stress event **(E)**. In both acclimation and memory state the clock will maintain its functions but very little is known on its possible phase shifts **(D,E)**. On the contrary, if the stress becomes exceptionally destructive, the clock will succumb, plants will not recover and die **(F)**. Solid arrows point to the most certain consequences of stress occurrence; dotted lines point to the possible consequences of recovery after stress; and dash/dotted lines point to the underlying mechanism of memory. Working clocks are coupled with identified physiological states of equilibrium while clock with the question marks represent the states where clock behavior is completely unknown. The red cross signifies the complete disruption of the clock when plants die. The yellow lightening bolt represents stress occurrence.

Cellular redox state and circadian rhythms influence one another continuously and diverse environmental stressors will most likely impact both traits. When plant cells are healthy and completely functional, cellular homeostasis is actively maintained, and plants are in a *dynamic equilibrium* with the environment (Strasser, 1988). Under this equilibrium, the relation between redox state and circadian rhythms is effective, with a functional clock resonating with the environmental cycles (**Figure 1A** – working clock). Any significant change in the environmental conditions triggers what is commonly known as ‘oxidative stress,’ a sudden change in the redox state compromising cellular homeostasis (Cramer et al., 2011). This state of *disequilibrium* seems to affect antioxidant enzymes gated by the clock (Lai et al., 2012), slowing scavenging activity and leading to elevated ROS levels (**Figure 1B** – unknown clock activity, question mark). Through a phase of *recovery* (**Figure 1C** – unknown clock activity, question mark), plants frequently reach a new *dynamic equilibrium* after stress exposure (**Figure 1D**). During recovery, RNA metabolism and post-transcriptional gene silencing appear to play key roles in resetting both the epigenome and transcriptome, but it remains unclear how the circadian clock responds at this stage (Crisp et al., 2016). ROS dynamics in the recovery phase are likewise ambiguous in plants (Einset et al., 2007; Ahuja et al., 2010; Zhang and Kay, 2010; Obata and Fernie, 2012), primarily because these molecules are highly reactive and have a short half-life (Ahmad et al., 2008; Suzuki et al., 2012). Quantifying ROS and antioxidant pools is challenging due to measurement artifacts and to the occurrence of small to moderate changes in some component pools with stress (Queval et al., 2008; Noctor et al., 2016). Protein- and metabolomics allow for consistent quantification of lipid and protein peroxidation or glutathione accumulation to estimate ROS-dependent changes (Kranner et al., 2006; Noctor and Foyer, 2016; Abdelrahman et al., 2017). However, fine-resolved transcriptomic data may be the best means to characterize the mechanistic response to ROS production and scrubbing under abiotic stress (Noctor et al., 2012).

There are potentially beneficial aspects to stress, for instance stimulating improved resistance to future stress (Larcher, 1980). A stress can be harmless if a plant manages to rapidly alter its homeostasis, adjusting metabolism, structure, and function to acclimate to altered conditions (**Figure 1D** – working clock) (Tsimilli-Michael et al., 1996). In both humans and rodents, stressors have been shown to lead to hormesis, appropriately priming organisms for future stress response (Foster and Kreitzman, 2014; Fleta-Soriano and Munné-Bosch, 2016). In the same manner, plants seem to retain a *memory* of the stress, improving their ability to respond to future variations in environmental conditions (Fleta-Soriano and Munné-Bosch, 2016) (**Figure 1E** – working clock). Possibly, RNA turnover contributes to acclimation and stress memory, but there is no clear understanding of how this mechanism competes with the epigenetics in memory development (Crisp et al., 2016). While clock function is retained, phase shifts are commonly associated with proximal stress response (**Figures 1D,E**). However, changes in clock gene frequencies can be also part of an adaptive evolutionary response, as in crop plants undergoing selection for agronomically desirable traits (Kevers et al., 2004; Yarkhunova et al., 2016). In short, after an initial destabilization, plants acclimate to the stress or improve their resistance to it, to the extent that physiological systems can buffer the changes in its redox state (**Figures 1A–E**). However, if the limits of plant tolerance are exceeded, then the stress becomes destructive, leading to permanent damage, loss of productivity or *death* (**Figure 1F** – absence of the clock).

While in rodents the connection between the circadian clock and stress response is well characterized (Koch et al., 2016), plant clock responses from early stress sensing to death, and its potential for re-setting after stress occurrence is still unclear (**Figures 1B,C**) (Grundy et al., 2015; Wulund and Reddy, 2015). Several stochastic models have successfully predicted clock activity at the molecular level in response to predictable variation in environmental cues (Ruoff et al., 2007; Resco et al., 2009; Domjian and Rand, 2011; Kosová et al., 2011). However, circadian clocks also experience extrinsic noise, namely irregular fluctuations in the environment, which are mostly omitted from current process models (Pokhilko et al., 2013; Guerriero et al., 2014; De Caluwé et al., 2016). Yet, synchronized metabolic responses driven by the core oscillator seem to be fundamental in plant response to environmental stress (Sanchez et al., 2011). Based on studies in mammals and algae, it seems probable that acute oxidative stress can reset the clock, resulting in the concurrent activation of a network of circadian genes that will propagate an antioxidant, cell survival response (Tamaru et al., 2013). This hypothesis considers H_2_O_2_ as a signal transducer, relaying information about the external environment to the circadian pacemaker. Susceptibility to oxidative stress through disruption of the circadian oscillator is another proposed mechanism linking ROS and the expression of the circadian clock (Qian et al., 2010).

We suggest more detailed studies during the recovery phase from the stress (**Figure 1C**). Recovery is not merely a return to the pre-stress state, but is instead a regulated mechanism, and its resolution would help in predicting plant adjustments to changing environmental conditions. Future research should focus on determining if ROS levels after a stress event may reset the periodicity of scavenger activity and affect clock gene expression (**Figure 1** – question marks). Moreover, it remains unclear how the clock behaves in extremely stressed plants close to mortality (Sanchez et al., 2011; Zhang et al., 2013; Resco de Dios and Gessler, 2017). In this scenario, a functional characterization of the effects of environmental noise on the core oscillator is key to integrating metabolic information, such as ROS dynamics, into current clock models (Einset et al., 2007; Miller et al., 2010; Zhang and Kay, 2010; Obata and Fernie, 2012; Haydon et al., 2013).

## Testing Clock/Redox State Interaction Under Stress Via Chlorophyll *a* Fluorescence

Chlorophyll *a* fluorescence is a fast, non-invasive method commonly used to assess plant performance (Baker, 2008; Croce and van Amerongen, 2014). The fluorescence signal (and its derived parameters) reliably mirror plant stress response under biotic and abiotic stress, for stress of different duration/intensity and across a variety of species, with higher responsiveness in light than dark conditions (Lichtenthaler et al., 1986; Baker and Rosenqvist, 2004; Woo et al., 2008; Papageorgiou and Govindjee, 2011). Although excessive ROS accumulation has been shown to occur together with changes in fluorescence parameters (Aldea et al., 2006; Moradi and Ismail, 2007) the direct mechanistic relation is still unclear (Gill and Tuteja, 2010). So far, no predictive understanding of the correlation between ROS and the fluorescence signal is possible and the association will depend on the stress type, intensity and duration.

**FIGURE 2:**
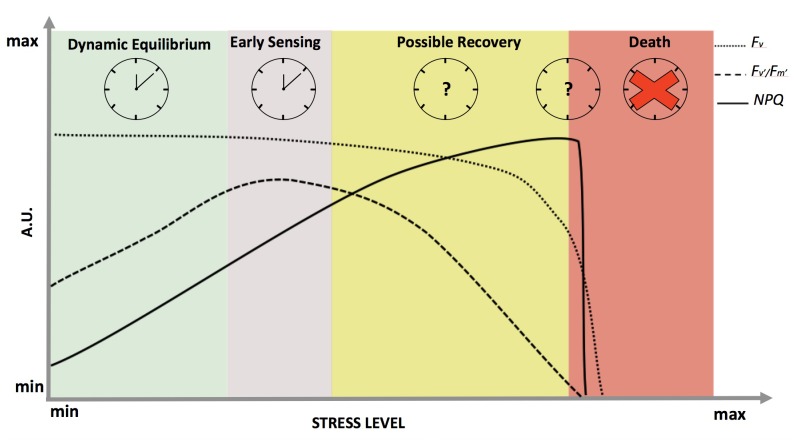
Dynamics of chlorophyll *a* fluorescence and derived parameters in response to abiotic stress in optimal light conditions. On the x-axis, the stress level is reported as a continuum between a minimum value at physiological equilibrium (green shadow), passing through early sensing (purple shadow) and possible recovery (yellow shadow), till death (red shadow). The y-axis represents fluorescence in arbitrary units: dotted, dashed and solid lines representing *F*_v_ (variable fluorescence signal), *F*_v_′*/F*_m_′ (maximum efficiency of photosystem II in light) and *NPQ* (Non-Photochemical Quenching), respectively. On the top part of the panel, working clocks are reported for the most studied physiological states while clock with the question marks represent the states where clock behavior is completely unknown. The red cross signifies the complete disruption of the clock when plants die.

In non-limiting light conditions, stress will differentially affect the variable fluorescence signal (*F*_v_) and fluorescence derived variables, such as the efficiency of photosystem II in light (*F*_v_′*/F*_m_′) and the Non-Photochemical Quenching (*NPQ*) (**Figure 2**). Each parameter reflects a specific aspect of photosynthetic activity, and in sum they depict a picture of the state of the photosynthetic light harvesting machinery (Roháček, 2002; Maxwell and Johnson, 2004). For the entire spectrum of stress response, *F*_v_ remains fairly constant (**Figure 2** – dotted line) until membrane failure at the cellular scale takes place, proximally leading to death of the plant after a distal cause such as severe drought (Guadagno et al., 2017). *NPQ* (**Figure 2** – solid line) is known to have a tight correlation with increasing stress (both for duration and/or intensity) (Müller et al., 2001; Demmig-Adams et al., 2014) until complete cellular failure at which point *F*_v_ will decline to zero and the plant is considered dead (Guadagno et al., 2017). *F*_v_′*/F*_m_′ seems to be the most reliable parameter to follow stress response dynamics from early sensing to mortality **(Figure 2** – dashed line). For instance, *F*_v_′*/F*_m_′ is able to capture the onset of the stress (*early sensing*) and increases to compensate for limited gas exchange due to stomatal closure with drought (Greenham et al., 2017). This is an informative outcome because in the past the theoretical maximum efficiency of PSII (*F*_v_*/F*_m_) has always been considered the main indicator of plant stress response (Murchie and Lawson, 2013). Typically, *F*_v_*/F*_m_ values decrease most significantly only under marked stress, when the survival of plants may have already been irretrievably reduced (Chen et al., 2015). On the other hand, *F*_v_′*/F*_m_′ dynamics more closely follow stress dynamics and the relative changes in redox state from early sensing to death in both conifers and herbaceous plants (Guadagno et al., 2017) **(Figure 2** – dashed line).

We emphasize the diurnal timing of plant response to abiotic stress can be critical, as we recently showed in *Brassica rapa* under mild drought stress (Des Mairas, 2017; Greenham et al., 2017). During the day, a phase shift in expression pattern for genes related to photosystem efficiency and light response pathways (e.g., *Light Harvesting Chlorophyll a/b Binding*-LHCB2.2, *Photosystem II Manganese Binding*-PSBY) was observed, consistent with the increase in *F*_v_′*/F*_m_′ (Greenham et al., 2017). Diel changes in chlorophyll *a* fluorescence were first shown in algae and phytoplankton (Prézelin and Ley, 1980; Sorek et al., 2013). Later, the same fluorescence parameters were observed to have rhythmicity in *Arabidopsis* mutants and barley under constant blue and white light conditions (Litthauer et al., 2015; Hussien et al., 2017), suggesting fluorescence as a possible high-throughput marker for circadian rhythms in plants as well as for changes in clock phase resulting from stress.

## Conclusion

During the last few years, several studies have confirmed circadian rhythms in redox state across species, suggesting the existence of a strong clock/redox interconnection. Although it seems clear that redox state and the circadian clock are interlocked in stress response, it remains unknown if the clock is reset by stress and if any type of protective acclimation is triggered at the cell level. In this perspective, we propose that a timely avenue of research lies in investigating the details of the recovery phase from the stress. We suggest a more intensive use of chlorophyll *a* fluorescence to assess variation in circadian rhythms, and summarized the importance of fluorescence dynamics at different stress levels. Fluorescence data as a high-throughput screen, coupled with ROS analysis, proteomic, metabolomics and gene expression, will inform and improve existing process models: acquiring realistic predictions for plant responses to a changing environment and ultimately improving breeding strategies.

## Author Contributions

CRG conceptualized, reviewed, validated formerly collected the data, and wrote the original draft of the manuscript. CRG and BEE worked on the visualization of the perspective. BEE and CW acquired the funding and reviewed and edited the manuscript.

## Conflict of Interest Statement

The authors declare that the research was conducted in the absence of any commercial or financial relationships that could be construed as a potential conflict of interest.
